# Supplementary material for: In Vitro Induction of Interspecific Hybrid and Polyploidy Derived from Oryza officinalis Wall
Source: Plants (Basel). 2023 Aug 20;12(16):3001. doi: 10.3390/plants12163001 (PMC10459038; doi:10.3390/plants12163001)
Supplement: Supplementary file 1 [file plants-12-03001-s001.zip › plants-2520152-supplementary-Figures.pdf]

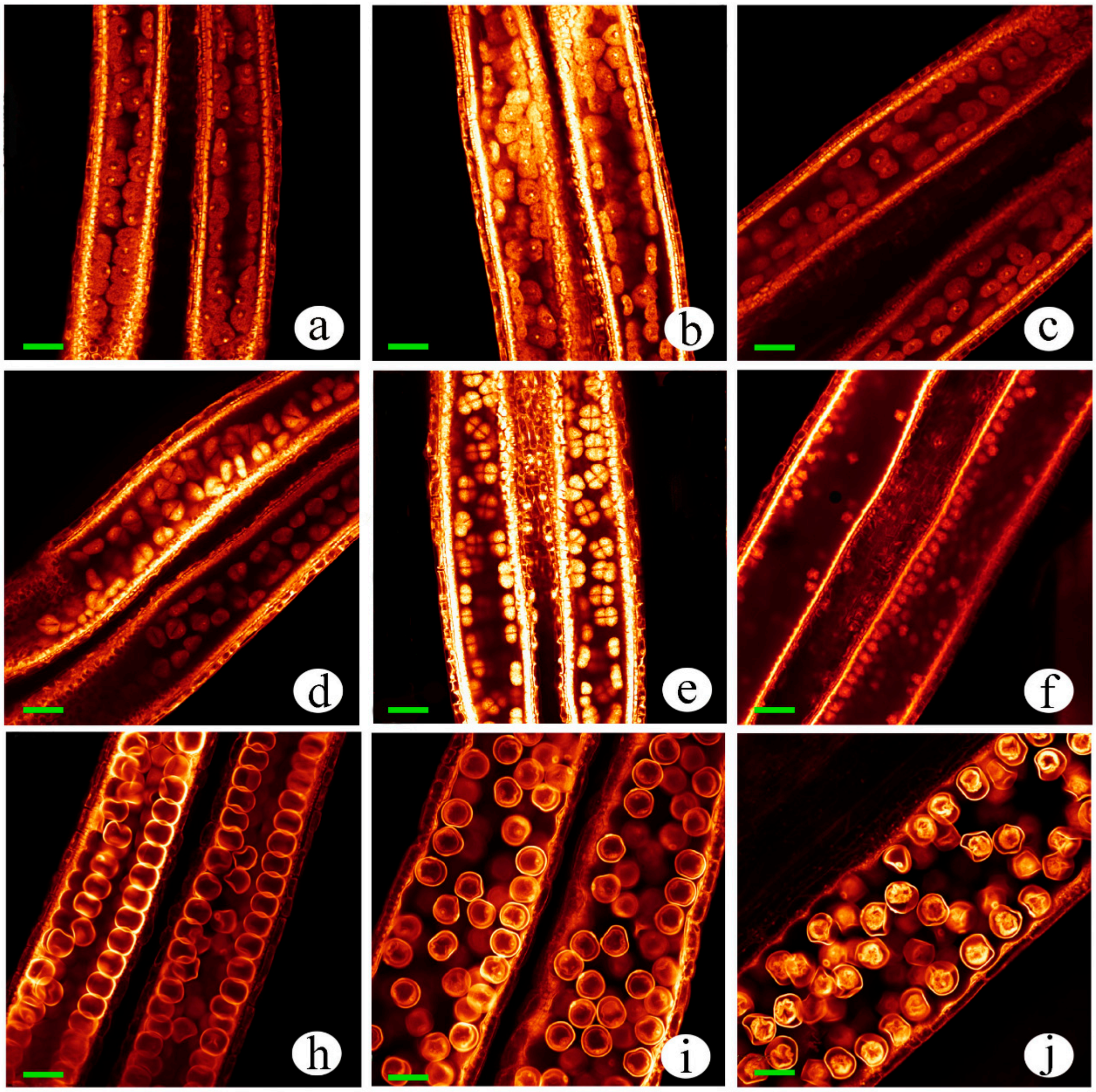

Figure S1. Normal pollen development of cultivar rice. a. Propahse I, pollen mother cells formation. b. Propahse I, pollen mother cells formation. c. Dyad stage. d. Tetrad stage. f. Single microspore stage. h. Middle bi-cellular pollen stage. i. Late bi-cellular pollen stage. j. Late bi-cellular pollen stage. Bars=40μm

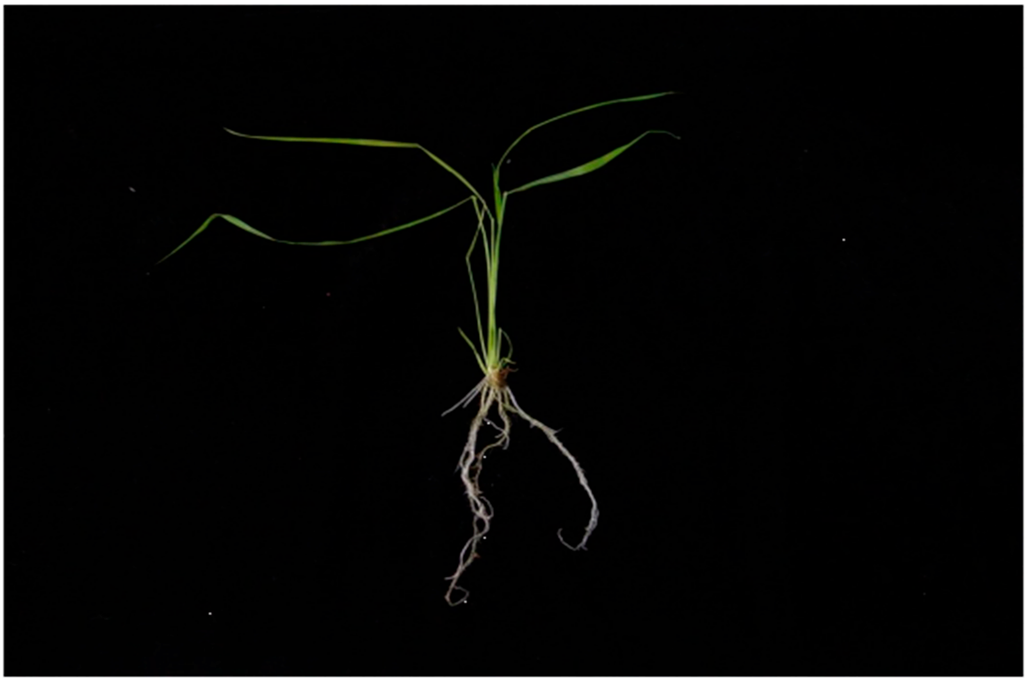

Figure S2. Tissue culture of strong rooting seedlings cultured for 20 days.

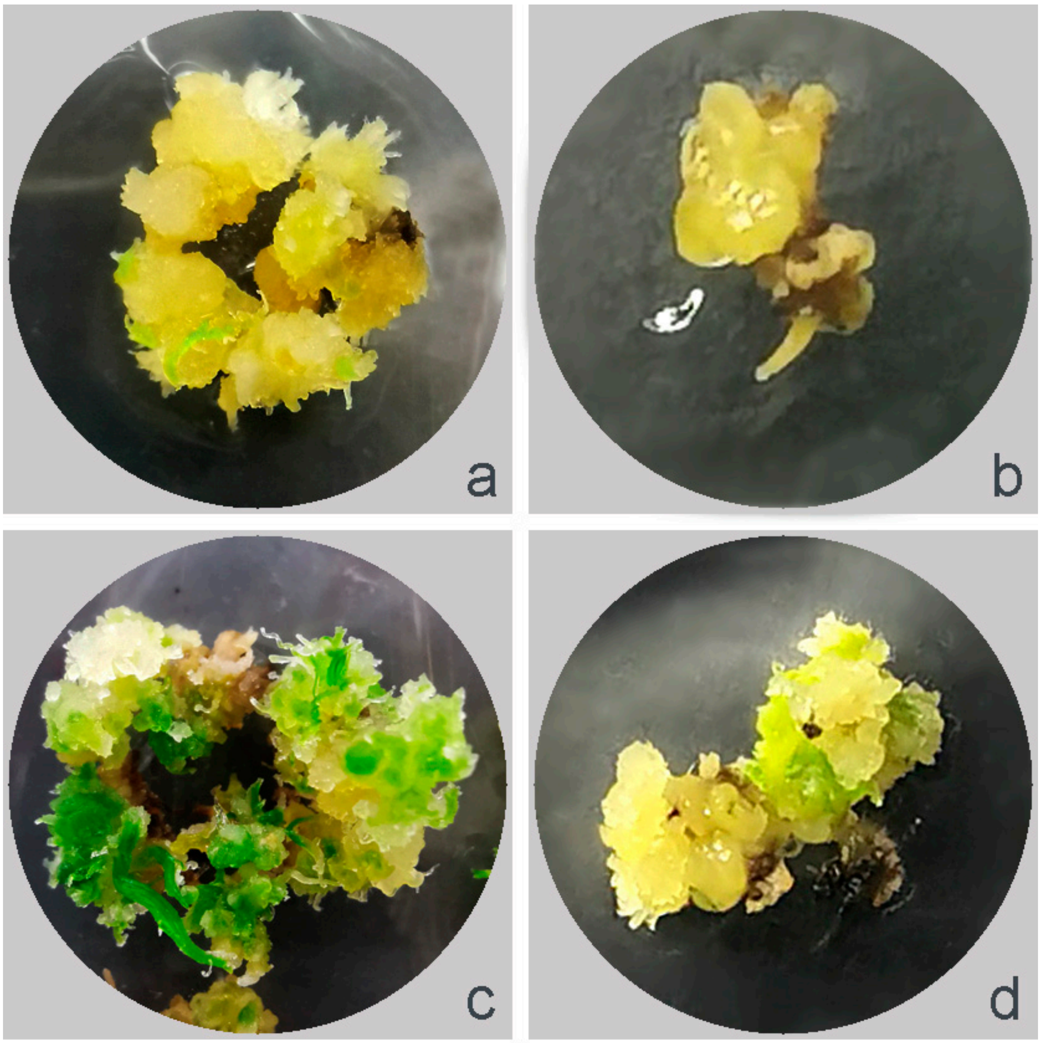

Figure S3. Callus differentiation at different durations in a 400 mg·L^{-1} colchicine co-culture. (a) and (c) Callus were co-cultured with colchicine 400 mg·L^{-1} for 3 days and then differentiated for 5 days and 20 days, respectively, and differentiated into young leaves. (b) and (d) Callus were co-cultured with 400 mg·L^{-1} colchicine for 5 days and then differentiated for 5 days and 20 days, respectively.
