# Supplementary material for: In Vitro Induction of Interspecific Hybrid and Polyploidy Derived from Oryza officinalis Wall
Source: Plants (Basel). 2023 Aug 20;12(16):3001. doi: 10.3390/plants12163001 (PMC10459038; doi:10.3390/plants12163001)
Supplement: Supplementary file 1 [file plants-12-03001-s001.zip › plants-2520152-supplementary-Tables.pdf]

Table S1. Tissue culture for callus differentiation

Combination Name	Medium Name	BA Concentration (mg·L^{-1})	KIN Concentration (mg·L^{-1})	NAA Concentration (mg·L^{-1})
F1	MS	2.0	0.0	0.4
F2	MS	2.0	0.0	0.2
F3	MS	0.0	2.0	0.2
F4	MS	2.0	2.0	1.0

Table S2. Effect of BA concentration on callus proliferation in the interspecific hybrid

Cultivation name	BA concentration ($mg \cdot L^{-1}$)	Seedling emergence rate (%)	Browning rate (%)
J1	0.0	821.92±74.59ab	3.75
J2	0.2	1036.43±200.98a	1.58
J3	0.5	617.46±122.5b	3.89

Note: Data in the results data table represented as mean±standard error (SE). Different lowercase letters represent significant differences when $P < 0.05$.

Table S3. Effect of colchicine co-culture on callus

Colchicine concentration ($mg \cdot L^{-1}$)	Processing time (d)	Treatment number	Survival rate (%)	Number of plants have	Seedling rate (%)
0（CK）	3	46	86.96	11	23.91
300	3	45	40.00	1	2.22
	5	50	26.00	0	0.00
400	3	45	68.89	6	13.33
	5	45	26.67	1	2.22
500	3	54	44.44	4	7.41
	5	54	22.22	0	0.00
600	3	62	14.52	1	1.61
	5	45	2.22	0	0.00
